# Is physiotherapy in migraines known to sufferers? A cross-sectional study

**DOI:** 10.1007/s10072-023-07195-9

**Published:** 2023-11-15

**Authors:** Roberto Tedeschi, Paolo Pillastrini, Giulia Pierangeli, Valentina Favoni, Pietro Cortelli, Sabina Cevoli

**Affiliations:** 1 Department of Biomedical and Neuromotor Sciences (DIBINEM), Alma Mater Studiorum, University of Bologna, Via Zamboni 33, 40126 Bologna, Italy; 2 Unit of Occupational Medicine, IRCCS Azienda Ospedaliero-Universitaria di Bologna, Bologna, Italy; 3 IRCCS Istituto Delle Scienze Neurologiche di Bologna, Bologna, Italy

**Keywords:** Migraine, Physiotherapy, Integrative treatment, Headache management, Migraine interventions

## Abstract

**Background:**

Migraine, a prevalent neurological condition, often impairs daily functioning and quality of life. While medications are the primary treatment, the potential of physiotherapy as an integrative approach remains underexplored. The aim of the study was to explore the awareness and experience of migraine patients regarding physiotherapy as a complementary treatment.

**Methods:**

A comprehensive survey was conducted on 200 migraine patients. Data collected included demographics, diagnosis, Migraine Disability Assessment Score Questionnaire (MIDAS) scores, and perceptions and experiences related to physiotherapy.

**Results:**

The average age of participants was 47.7 ± 13.2 years, with a predominance of females, 149 out of 200 (74.5%). The mean MIDAS score was 36.7 ± 45.3, indicating a significant impact on daily life. While 39 out of 200 (19.5%) had undergone physiotherapy for their headache, 161 out of 200 (80.5%) had not. Of those who had, 22 out of 39 (56.4%) reported benefits, including reduced attack intensity and frequency. Interestingly, 145 out of 161 (90.1%) expressed interest in physiotherapy, with many expecting it to reduce attack intensity, 57 out of 200 (28.5%) and frequency, 77 out of 200 (38.5%).

**Conclusions:**

The study highlights the substantial burden of migraines and the potential of physiotherapy as an adjunctive treatment. Increasing awareness and accessibility to physiotherapy could offer migraine patients a more holistic treatment approach; however, randomized controlled trials are mandatory in order to confirm its efficacy.

**Supplementary Information:**

The online version contains supplementary material available at 10.1007/s10072-023-07195-9.

## Introduction

Headache stands as one of the most prevalent clinical disorders globally, influencing the daily lives of millions of individuals [1–3]. Characterized by a wide range of symptoms and causes, headaches can manifest in various forms, including migraine, tension-type headache, and cluster headache [4–6]. These forms of headache, particularly migraine, have a significant impact on patients’ quality of life, limiting their daily activities and increasing their reliance on medical treatments [7]. Migraine, in particular, is a chronic neurological condition that manifests through intense, throbbing headache attacks, often accompanied by nausea, photophobia, and phonophobia [8, 9]. Its pathophysiology is complex, involving various neurological and vascular mechanisms, particularly the trigeminovascular system [10]. Despite its prevalence and societal impact, a full understanding of migraine and its underlying mechanisms remains an active area of research [11]. Within the context of migraine treatment, physiotherapy emerges as a potential complementary therapeutic modality [12–15]. Physiotherapy, through manual techniques, exercises, and behavioral interventions, can offer relief to patients, reducing the frequency and intensity of migraine attacks [16, 17]. However, the awareness and adoption of physiotherapy as a treatment for migraine vary significantly among patients [16]. This study aims to explore the awareness and experience of migraine patients regarding physiotherapy as a complementary treatment. Through a detailed investigation, it intends to better understand how patients perceive physiotherapy, whether they have been exposed to such treatment, and what their expectations and experiences are concerning it. With this information, it is hoped to provide a solid foundation for future trials in order to validate new therapeutic interventions and awareness strategies, aimed at improving the quality of life of migraine patients.

## Methods

### Study design and population

This is a cross-sectional observational study. No therapeutic intervention was administered during the study, and all procedures adhered to standard clinical practice. The study population comprised migraine patients attending the Centro per lo Studio e la Cura delle Cefalee ed Algie Facciali in Bologna, either for an initial consultation or a routine follow-up. All participants had a confirmed diagnosis of migraine for a minimum of 3 months, as determined by headache specialists. The study received approval from our institutional review board. All participants provided informed consent in accordance with our institution’s data collection and disclosure policy. Further ethical review was deemed unnecessary as no personally identifiable information was collected or stored.

### Recruitment

#### Inclusion criteria

1. Patients diagnosed with episodic migraine without aura, chronic migraine, or migraine with aura (according to the criteria of the International Headache Classification, ICHD-3 [18]).
2. The participant is willing and able to give consent to participate in the study.
3. Male or female, aged ≥ 18 years.

#### Exclusion criteria

1. Age < 18 years.
2. The participant is unwilling and/or unable to give consent to participate in the study.

#### Recruitment procedure

Patients presenting for an initial visit or a check-up were informed about the study and asked if they wished to participate. After providing informed consent, patients were interviewed using a semi-structured interview lasting approximately 5 min (MINDS Questionnaire—MigraIne aND PhySiotherapy is provided as a supplementary file to this article (Supplementary File 1).

During the interview, demographic data such as age, gender, occupation, migraine frequency, localization of the pain, and any sports activities practiced were collected. Additionally, the score from the Migraine Disability Assessment Score Questionnaire (MIDAS) [19] questionnaire, which is regularly administered during routine visits, was recorded. Recruitment was continuous and consecutive, and patients were free to join or decline participation in the study without any pressure or incentive. All collected data will be treated with the utmost confidentiality and in compliance with data protection regulations.

### Outcomes

The primary and secondary outcomes are as follows:

## Primary outcomes:

- **Awareness level**: Determine the percentage of migraine patients who are aware of physiotherapy as a potential treatment option for their condition.
- **Previous experience**: Ascertain the number of participants who have previously undergone physiotherapy for migraine relief and their overall satisfaction with the treatment.
- **Beneficial outcomes**: Evaluate the reported benefits from those who have undergone physiotherapy, including:
  ○ Reduction in the frequency of migraine attacks.
  ○ Decrease in the intensity of migraine episodes.
  ○ Reduction in medication intake.
  ○ Improvement in overall quality of life.

## Secondary outcomes:

- **Recommendation source**: Identify the primary sources (e.g., general practitioners, neurologists, friends, or self-research) that have recommended physiotherapy to the participants.
- **Interest in future physiotherapy**: Gauge the level of interest among participants in trying physiotherapy as a potential treatment option, especially among those who haven’t tried it before.
- **Expectations**: Understand the primary expectations of patients when considering physiotherapy for migraine relief. This includes their hopes regarding the reduction in attack frequency, intensity, medication intake, and overall improvement in life quality.

### Statistical analyses

Considering the enrollment rate, we decided to consecutively enroll a total of 200 subjects, from May 2022 to January 2023. Frequencies and percentages will be used to summarize categorical variables such as awareness of physiotherapy, previous experiences with physiotherapy, and responses to specific questions in the MINDS questionnaire. Mean, median, standard deviation, and range will be used for continuous variables like age, gender, and MIDAS scores.

## Results

In our study of 200 participants, the demographic landscape consisted mainly of middle-aged individuals, with a mean age of 47.7 years (SD = 13.2). The youngest participant was 19 years old, while the oldest was 90, and the mode was 40 years (Table 1).

**Table 1:** Demographic characteristics

	All sample (*n* = 200)
Age (years)	47.7 ± 13.2
Gender	
Men	51 (25.5%)
Women	149 (74.5%)
Education	
Primary school Secondary school High school Degree	6 (3.0%) 21 (10.5%) 92 (46.0%) 81 (40.5%)
Work activities	
Employee Professional Retiree Student Homemaker Unemployed Worker	103 (51.5%) 45 (22.5%) 21 (10.0%) 1 (0.5%) 3 (1.5%) 16 (8.0%) 11 (5.5%)
Sport activities	
Absent Low Moderate High	50 (25.0%) 83 (41.5%) 59 (29.5%) 8 (4.0%)

In the “Sport activities” section, I have included the requested descriptions for the different levels of physical activity: “Absent” (0 physical activity sessions per week), “Low” (1 physical activity session per week), “Moderate” (2–3 physical activity sessions per week), and “High” (more than 3 physical activity sessions per week)

Gender distribution was skewed towards females, who constituted nearly three-quarters of the sample 74.5%. In terms of educational attainment, a significant proportion had completed high school (46.0%) or pursued a degree (40.5%). Only a small fraction had their education limited to primary school (3.0%) (Table 1).

Looking at work-related activities, more than half of the participants were employed (51.5%). Professionals, including those in specialized fields, made up 22.5% of the sample. Retired people were 10.5%, the unemployed 8.0%, and students were 0.5% of the sample (Table 1).

Sporting activities varied among participants. A considerable 41.5% engaged in low-intensity sports, while 29.5% opted for moderate-intensity activities. Those who refrained from sports altogether accounted for 25.0%, and a 4.0% indulged in high-intensity sports (Table 1).

Turning our attention to the diagnostic data, episodic medium- to high-frequency migraine without aura was the predominant diagnosis, encompassing 48.0% of the sample. Chronic migraine was identified in 31.0%, and episodic low-frequency migraine without aura in 17.0%. Migraine with aura was diagnosed in 4.0% of participants (Table 2).

**Table 2:** Prevalence of migraine diagnoses, presence of occipital-cervical pain, and contraindications to physiotherapy in the sample population

	All sample (*n* = 200)
Diagnosis	
Chronic migraine Episodic low-frequency migraine without aura Episodic medium- to high-frequency migraine without aura Migraine with aura	62 (31.0%) 34 (17.0%) 96 (48.0%) 8 (4.0%)
Occipital-cervical pain	
Yes No	168 (34.0%) 132 (66.0%)
Contraindications to physiotherapy	
Yes No	197 (98.5%) 3 (1.5%)
MIDAS (Migraine Disability Assessment Score Questionnaire)	36.7 ± 45.3

Low-frequency migraine: refers to individuals experiencing fewer than 4 migraine attacks per month, medium-frequency migraine: denotes those with between 4 and 14 migraine attacks per month, chronic migraine: characterized by individuals having more than 15 migraine attacks per month

The average score of MIDAS stood at 36.7, with a standard deviation of 45.3.

The prevalence of occipito-cervical pain was reported by 34.0% of the sample. With regard to physiotherapy, 98.5% had no contraindications, while only a small 1.5% had contraindications (Table 2).

In Table 3, which delves into participants’ prior experiences with physiotherapy, a significant 80.5% revealed they had never sought physiotherapy for their headache. Among the 19.5% who had, a notable 56.4% acknowledged its benefits, especially highlighting a decrease in the frequency and severity of their migraine attacks. Notably, of these, 18 participants reported experiencing occipital pain. Manual therapy was predominantly recognized as the most common type of physiotherapy received.

**Table 3:** Participant responses to key questions from the MINDS Questionnaire on physiotherapy and migraine management

MINDS Questionnaire Question	Frequencies (Yes)	Percentage (Yes) (%)	Main outcomes (%)
Have you ever undergone physiotherapy for your headache?	39	19.5%	-
Did you benefit from it?	22	56.4%	-
In what way?	-	-	Reduction in attack intensity (36.4%), reduction in the number of attacks (31.8%), reduction of drug intake (18.2%), improved quality of life/reduced disability due to headache (9.1%), others (4.5%)
Do you remember what type of physiotherapy?	-	-	Manual therapy (82.1%), physical therapy (10.3%), exercises (7.7%)
Have you ever been suggested to undergo physiotherapy?	10	6.2%	-
Are you interested?	145	90.1%	-
What kind of treatment do you think they would propose to you in a course of physiotherapy?	-	-	Massages (44.4%), manipulations (27.2%), exercises (13.0%), others (11.7%), physical therapies (3.7%)
What would you expect from a course of physiotherapy?	-	-	Reduction in the number of attacks (38.5%), reduction in attack intensity (28.5%), improved quality of life/reduced disability due to headache (24.0%), reduction of drug intake (8.0%), others (1.0%)

Though a vast majority (93.8%) had never been advised to consider physiotherapy, a 90.1% expressed a keen interest in exploring it. When asked about their expectations from such treatments, massages (44.4%) and manipulations (27.2%) were the most anticipated interventions. Overall, the primary aspiration from physiotherapy among participants was to achieve a tangible reduction in both the number (38.5%) and intensity (28.5%) of migraine episodes.

## Discussion

The management of migraine, a debilitating condition that profoundly impacts daily functioning and quality of life, has traditionally been centered around pharmacological interventions [20]. However, as the medical community seeks more holistic approaches to patient care, the role of physiotherapy as an integrative treatment for migraine has come to the forefront [13, 21]. Our study aimed to illuminate this aspect, exploring patients’ awareness, perceptions, and experiences related to physiotherapy for migraine management. We observed an elevated MIDAS scores in our study cohort, underscoring the severe disability and disruption migraines can cause in an individual’s life, from missed workdays to reduced participation in social and familial activities [19, 22]. This first aspect emphasizes the pressing need for effective and comprehensive migraine management strategies. One of the salient findings of our research was the evident gap in patients’ awareness of physiotherapy as a viable treatment option for their headaches. A significant majority had never undergone physiotherapy for this ailment. However, it’s noteworthy to highlight that despite this lack of exposure, an overwhelming 90% expressed a keen interest in pursuing physiotherapy or gaining more knowledge about it. This underscores a potential untapped avenue for migraine management and emphasizes the need for better patient education and awareness campaigns regarding alternative and complementary therapeutic options. MIDAS scores underscore the need for enhanced patient education and advocacy, ensuring that individuals are informed about the full spectrum of available treatments. A concerning observation from our study was the lack of regular physical activity among participants. A significant 66.5% either refrained from sports altogether or engaged in them only sporadically. This lack of consistent physical activity further underscores the importance of exploring physiotherapy as a complementary approach to traditional treatments. The occipital region is innervated by the upper cervical roots, which are also implicated in the pathophysiology of cervicogenic headaches, a common comorbidity or misdiagnosis in patients with migraines [23, 24]. For those who had experienced physiotherapy, the benefits were palpable. A majority reported tangible improvements, particularly in terms of reduced attack intensity and frequency. This aligns with the growing body of evidence that underscores the probable effectiveness of physiotherapy in migraine management [15, 25, 26]. Physiotherapy emerges as a potential integrative tool in the treatment of migraine, with techniques such as manual therapy, which was the most recalled in our study, demonstrating to alleviate migraine symptoms, possibly addressing musculoskeletal dysfunctions that can trigger or exacerbate headaches, including those stemming from cervical pain [26–28]. Patients’ expectations from physiotherapy, as revealed in our study, were predominantly centered on tangible outcomes: a reduction in the number and intensity of migraine attacks, highlighting the urgent need for relief among migraine sufferers and reinforcing the potential role of physiotherapy in meeting these expectations. However, it is crucial to underscore the low methodological quality of existing studies, which often merge migraine and tension-type headache without clear distinctions, and the lack of controlled studies with adequate power. This deficiency is what contributes to the scant consideration of physiotherapy and its limited prescription in the clinical context. The necessity to conduct controlled and randomized trials is therefore imperative to solidify the position of physiotherapy in the therapeutic panorama of migraine. The possible role of physiotherapy in migraine might be rooted in a solid pathophysiological basis. The connection between the trigeminal and cervical nervous systems [29], in particular, provides a plausible explanation for why physiotherapy, and manual therapy in particular, can be effective in treating migraines [30]. The trigeminal-cervical system plays a key role in pain perception in the head and neck region. Nociception from the cervical muscles and encephalic dura mater converges on brainstem neurons, creating an interaction between painful signals from cranial and cervical structures [31]. This convergence of painful inputs can facilitate central sensitization and contribute to the onset and persistence of migraines. Physiotherapy, through techniques such as manual therapy, can aim to modulate this pain [32] and reduce muscle tension in the cervical region, potentially positively influencing the trigeminal-cervical system and, consequently, mitigating migraine symptoms. Furthermore, the physiotherapeutic approach can also aim to improve posture and neck mechanics, which can be additional contributing factors to the chronicization of pain in individuals with migraines. It is essential to consider neck pain not only as a symptom but also as a potential causal factor in migraine pathophysiology, which can be effectively managed through targeted physiotherapeutic interventions [26–28].

## Conclusions

Migraine, a condition that significantly impairs daily functioning and quality of life, necessitates a comprehensive approach to management. Our study has highlighted the potential of physiotherapy as an integrative treatment option, yet also revealed a notable gap in patient awareness. While traditional pharmacological treatments remain essential, the tangible benefits reported by those who underwent physiotherapy emphasize its potential role in reducing migraine intensity and frequency. As the medical landscape shifts towards a more holistic approach to patient care, it becomes essential to advocate for diverse therapeutic options, including physiotherapy.

### Supplementary Information

Below is the link to the electronic supplementary material.

Supplementary file1 (DOCX 15 KB)

## Data Availability

The datasets generated and/or analyzed during the current study are not publicly available due to privacy and ethical considerations but are available from the corresponding author on reasonable request.

## References

[1] Nye BL, Ward TN (2015). Clinic and emergency room evaluation and testing of headache. Headache.

[2] Schwedt TJ (2014). Chronic migraine. BMJ.

[3] Torres-Ferrús M, Ursitti F, Alpuente A (2020). From transformation to chronification of migraine: pathophysiological and clinical aspects. J Headache Pain.

[4] Garagnani P, Terlizzi R, Cevoli S (2015). Genomics and epigenomics. J Headache Pain.

[5] Aurora SK (2009). Is chronic migraine one end of a spectrum of migraine or a separate entity?. Cephalalgia.

[6] Natoli JL, Manack A, Dean B (2010). Global prevalence of chronic migraine: a systematic review. Cephalalgia.

[7] Covelli V, Guastafierro E, Raggi A (2018). The evaluation of difficulties with work-related activities caused by migraine: towards a specific questionnaire. Neurol Sci.

[8] Bigal ME, Serrano D, Buse D (2008). Acute migraine medications and evolution from episodic to chronic migraine: a longitudinal population-based study. Headache.

[9] D’Amico D, Usai S, Grazzi L (2003). Quality of life and disability in primary chronic daily headaches. Neurol Sci.

[10] Bigal ME, Serrano D, Reed M, Lipton RB (2008). Chronic migraine in the population: burden, diagnosis, and satisfaction with treatment. Neurology.

[11] Lipton RB (2009). Tracing transformation: chronic migraine classification, progression, and epidemiology. Neurology.

[12] Luedtke K, Allers A, Schulte LH, May A (2016). Efficacy of interventions used by physiotherapists for patients with headache and migraine-systematic review and meta-analysis. Cephalalgia.

[13] Beier D, Callesen HE, Carlsen LN (2022). Manual joint mobilisation techniques, supervised physical activity, psychological treatment, acupuncture and patient education in migraine treatment. A Syst Rev Meta-Anal Cephalalgia.

[14] Chaibi A, Tuchin PJ, Russell MB (2011). Manual therapies for migraine: a systematic review. J Headache Pain.

[15] Bialosky JE, Bishop MD, Price DD (2009). The mechanisms of manual therapy in the treatment of musculoskeletal pain: a comprehensive model. Man Ther.

[16] G C, R Q, K L (2023) Migraine patients’ experiences with and expectations from physiotherapy. Musculoskelet Sci Pract 66. 10.1016/j.msksp.2023.102803 10.1016/j.msksp.2023.102803 37331925

[17] Bevilaqua-Grossi D, Pinheiro-Araujo CF, Carvalho GF, Florencio LL (2023). Neck pain repercussions in migraine - the role of physiotherapy. Musculoskelet Sci Pract.

[18] (2018) Headache Classification Committee of the International Headache Society (IHS) The International Classification of Headache Disorders, 3rd edition. Cephalalgia 38:1–211. 10.1177/0333102417738202 10.1177/0333102417738202 29368949

[19] Stewart WF, Lipton RB, Dowson AJ, Sawyer J (2001). Development and testing of the Migraine Disability Assessment (MIDAS) Questionnaire to assess headache-related disability. Neurology.

[20] Guitera V, Muñoz P, Castillo J, Pascual J (2002). Quality of life in chronic daily headache: a study in a general population. Neurology.

[21] Castien R, De Hertogh W (2019). A neuroscience perspective of physical treatment of headache and neck pain. Front Neurol.

[22] Carvalho GF, Luedtke K, Braun T (2021). Minimal important change and responsiveness of the Migraine Disability Assessment Score (MIDAS) questionnaire. J Headache Pain.

[23] Sollmann N, Schandelmaier P, Weidlich D (2023). Headache frequency and neck pain are associated with trapezius muscle T2 in tension-type headache among young adults. J Headache Pain.

[24] Messina R, Cetta I, Colombo B, Filippi M (2022). Tracking the evolution of non-headache symptoms through the migraine attack. J Headache Pain.

[25] Schmid A, Brunner F, Wright A, Bachmann LM (2008). Paradigm shift in manual therapy? Evidence for a central nervous system component in the response to passive cervical joint mobilisation. Man Ther.

[26] Onan D, Ekizoğlu E, Arıkan H (2023). The efficacy of physical therapy and rehabilitation approaches in chronic migraine: a systematic review and meta-analysis. J Integr Neurosci.

[27] Williamson TJ, Bolles CL, Hedges NA, Kettner NW (2021). Chronic primary pain of the spine: an integrative perspective Part 1. SN Compr Clin Med.

[28] Williamson TJ, Bolles CL, Hedges NA, Kettner NW (2021). Chronic primary pain of the spine: an integrative perspective Part 2. SN Compr Clin Med.

[29] Rist PM, Hernandez A, Bernstein C (2019). The impact of spinal manipulation on migraine pain and disability: a systematic review and meta-analysis. Headache.

[30] Carvalho GF, Schwarz A, Szikszay TM (2020). Physical therapy and migraine: musculoskeletal and balance dysfunctions and their relevance for clinical practice. Braz J Phys Ther.

[31] Fernández-de-Las-Peñas C, Cuadrado ML (2016). Physical therapy for headaches. Cephalalgia.

[32] Han X, Yu S (2023). Non-pharmacological treatment for chronic migraine. Curr Pain Headache Rep.

