# Supplementary material for: Is physiotherapy in migraines known to sufferers? A cross-sectional study
Source: Neurol Sci. 2023 Nov 15;45(4):1669–74. doi: 10.1007/s10072-023-07195-9 (PMC10942894; doi:10.1007/s10072-023-07195-9)
Supplement: Supplementary file 1 — Supplementary file1 (DOCX 15 KB) [file 10072_2023_7195_MOESM1_ESM.docx]

**MINDS Questionnaire - MigraIne aND PhySiotherapy**

The MINDS questionnaire is designed to assess the awareness and experiences of migraine patients regarding physiotherapy as a potential treatment. It consists of the following sections:

1. Previous Experience with Physiotherapy:
   - Question: "Have you ever undergone physiotherapy for your headache?"
     - Possible answers: Yes / No
   - (If the answer is "Yes") Question: "Do you remember the type of physiotherapy and who suggested it to you?"
     - Open-ended response to specify the type of physiotherapy and the source of the suggestion.
2. Benefits from Physiotherapy:
   - Question: "Did you benefit from it?"
     - Possible answers: Yes / No
   - (If the answer is "Yes") Question: "In what terms did you find benefits?"
     - Answer options:
       - Reduction in the number of attacks
       - Reduction in the intensity of attacks
       - Reduced medication intake
       - Improved quality of life/reduction in disability due to headaches
       - Other (with space for an open-ended response)
3. Proposal for Physiotherapy:
   - (If they have never undergone physiotherapy) Question: "Has it ever been suggested to you?"
     - Possible answers: Yes / No
   - Question: "Would you be interested in a physiotherapy cycle?"
     - Possible answers: Yes / No
   - Question: "What treatment do you think they would suggest in a physiotherapy cycle?"
     - Open-ended response to specify the patient's expectations.
4. Expectations from Physiotherapy:
   - Question: "What would you expect from a physiotherapy cycle?"
     - Answer options:
       - Reduction in the number of attacks
       - Reduction in the intensity of attacks
       - Reduced medication intake
       - Improved quality of life/reduction in disability due to headaches
       - Other (with space for an open-ended response)
